# Efficacy and Safety of Intraoperative Lumbar Drain in Endoscopic Skull Base Tumor Resection: A Meta-Analysis

**DOI:** 10.3389/fonc.2020.00606

**Published:** 2020-05-07

**Authors:** Xiaoming Guo, Yueli Zhu, Yuan Hong

**Affiliations:** ^1^Department of Neurosurgery, Second Affiliated Hospital, School of Medicine, Zhejiang University, Hangzhou, China; ^2^Department of Neurology, Second Affiliated Hospital, School of Medicine, Zhejiang University, Hangzhou, China

**Keywords:** cerebrospinal fluid leak, skull base tumor, lumbar drainage, endoscopic endonasal surgery, pituitary

## Abstract

**Objectives:** This study aims to evaluate the efficacy and safety of lumbar drainage (LD) in preventing cerebrospinal fluid (CSF) leaks after endoscopic skull base tumor resection.

**Methods:** A systematic online search was conducted using PubMed, Embase, Scopus, Web of Science, and Cochrane Library from January 2006 to July 2019. Data analyses were performed by the Cochrane Collaboration's Review Manager 5.3 software.

**Results:** Eight studies, including two randomized controlled trials and six observational studies, met the inclusion criteria. No significant difference was found in the post-operative CSF leak rate between the LD group and the non-LD group [odds ratio (OR), 0.80; 95%CI, 0.37–1.74; *I*^2^ = 37%; *P* = 0.57). Subgroup analysis of the intraoperative high-flow leaks, including 4 studies and 313 patients, showed that LD was associated with reduced likelihood of post-operative CSF leak (OR, 0.37; 95%CI, 0.17–0.83; *I*^2^ = 0%; *P* = 0.02). The placement of LD was related to increased risk of headache compared with non-LD use, and no significant difference was found in the occurrence of deep vein thromboses and pulmonary emboli between two groups.

**Conclusion:** LD is not recommended in all patients undergoing endoscopic skull base tumor resection. However, for patients with intraoperative high-flow leaks, LD is effective and safe in reducing risk of CSF leak.

## Introduction

The endoscopic endonasal approach is a safe and effective surgical technique in the resection of skull base lesions. However, proper skull base reconstruction to prevent the occurrence of post-operative cerebrospinal fluid (CSF) leakage remains a major challenge following these operations (1, 2). The lumbar drainage (LD) is a practice in the management of CSF leaks after endoscopic skull base tumor resection. This device is often kept in place preoperatively or post-operatively to reduce intracranial pressure by continuous drainage, which is believed to facilitate healing of the dural repair under decreased tension and lower the possibility of persistent CSF fistula (3–5). In addition, LD can be conversely used to add saline into the lumbar cistern to provoke descent of skull base tumors, such as pituitary adenomas.

The high-flow leak, which was defined as entrance into an arachnoid cistern or ventricle, is more challenging to deal with (6). Preoperative LD is of particular importance when a high-flow leak is encountered during the procedure. In 2006, the nasoseptal flaps (NSFs) were introduced by Hadad et al. (7). The overall rate of post-operative CSF leak dramatically reduced from 40 to 5% (7, 8). With the increased dependability of the NSFs for skull base reconstructions, some studies reported that LD is being overused in endoscopic skull base surgery when modern reconstructive techniques are used, even when there is a high-flow leak (3, 9). Furthermore, there is little consensus on the use of LD in endoscopic skull base surgery, including identifying suitable patients for LD placement and the duration of LD placement (3, 10). Given the potential side effects including headache, radiculopathy, overdrainage, and decreased patient mobilization, the use of LD has become controversial (11).

Previous studies have investigated the role of LD on the onset of post-operative CSF leaks, but the results have been controversial (10, 12, 13). Therefore, the purpose of our meta-analysis is to explore whether adjunct LD can reduce the rate of post-operative CSF leak in patients undergoing endoscopic skull base surgery and to further find out factors that may contribute to post-operative CSF leaks.

## Methods

This meta-analysis was performed according to the Preferred Reporting Items for Systematic Reviews and Meta-Analyses (PRISMA) guidelines (14).

### Search Strategy

A comprehensive search strategy included the terms: “lumbar drain,” “CSF diversion,” “skull base tumor,” and “endoscopic endonasal surgery” with appropriate synonyms. PubMed, Embase, Scopus, Web of Science, and Cochrane Library were screened for eligible studies. In light of the substantial advances in techniques and materials with the adoption of the NSFs and other pedicled vascularized tissue flaps used in reconstruction of skull base surgeries, searches were limited from January 2006 to June 2019. We also manually searched the references cited in clinical trial reports or reviews to identify additional relevant studies (Supplementary Data Sheet 1).

### Eligibility and Exclusion Criteria

We included all research articles published in English that met all of the following criteria: (i) studies should be randomized controlled trials (RCTs) or observational studies; (ii) LD must be placed at the beginning or at the end of the surgical procedure; (iii) LD must maintain into the early post-operative period; (iv) studies were required to use multilayered repair strategy with NSFs for reconstruction; (v) studies must specify that CSF leaks were secondary to endoscopic skull base tumor resection; (vi) studies must contain two arms, LD group and non-LD group; and (vii) studies were required to have reported the number of patients, number of cases with intraoperative LD placement, and the number of cases with post-operative CSF leaks in LD group and non-LD group.

Studies were excluded if they met any of the criteria: (i) studies included patients that underwent open, combined open, and endoscopic or microscopic approaches; (ii) all the articles analyzed about preoperative CSF leaks that resulted from traumatic-, idiopathic-, or surgery-related iatrogenic causes; (iii) studies that did not provide the number of cases with the placement of LD or the number of post-operative CSF leaks in both groups; and (iv) case reports, review articles, editorials clinical guidelines, and unpublished studies (e.g., conference abstracts).

Eligible studies were screened by two independent investigators (XG and YZ). All disagreements were resolved by a third reviewer (YH).

### Data Extraction and Outcomes

Relevant data was extracted independently from each study using a standardized form by two investigators (XG and YZ). We extracted the following information from each study: general information (first author's name, year of publication, and location), details of study design, patients' characteristics (including gender, age, BMI), sample size, LD placement protocol, reconstruction strategy, lesions location (anterior fossa, sellar/suprasellar, and posterior fossa), pathological type (i.e., pituitary adenoma), number of cases with intraoperative LD placement, and the number of high-flow intraoperative leaks (when available), number of adverse events (AEs, when available), and post-operative CSF leaks with or without intraoperative LD placement. The primary outcome was the rate of post-operative CSF leak with or without pre- or intraoperative LD placement. Postoperative CSF leaks were determined by clinical evidence of CSF rhinorrhea. The secondary outcome was the rate of AEs that were recorded separately. All disagreements were resolved by a third reviewer (YH).

### Risk of Bias Assessment

Two investigators (XG and YZ) independently assessed the risk of bias for the included RCTs using the Cochrane risk of bias tool (15). This tool includes the following domains for methodological evaluation: (i) sequence generation; (ii) allocation concealment; (iii) blinding of participants, personnel, and outcome assessors; (iv) incomplete outcome data; (v) selective outcome reporting; and (vi) other sources of bias. The RCT was ranked as low risk of bias (low risk of bias for all domains), high risk (high risk of bias for one or more domains), or unclear risk (unclear risk of bias for one or more key domains). For observational studies, we used the Newcastle–Ottawa Scale (NOS) (16). The criteria included selection of the exposed/unexposed cohort, comparability of the study group, and the outcome assessment. Studies with a total score of 6 or more were defined as high quality. Publication bias was assessed using a funnel plot. When the shape of the funnel plot was asymmetric, possible publication bias was determined.

### Statistical Analysis

Statistical analyses were performed using the Review Manager 5.3 software (The Nordic Cochrane Center, The Cochrane Collaboration, Copenhagen). The odds ratio (OR) was used to assess the association between LD use and risk of CSF leak. We performed this meta-analysis under the random-effects model to pool OR with 95% confidence interval (CI) for the incidence of CSF leak. We further analyzed the results in studies classified by several factors (such as the flowrate of intraoperative leak, study design, and pathological type) to explore important clinical differences. The degree of heterogeneity was estimated by *I*^2^. An *I*^2^ value <25% indicated low heterogeneity, a value between 25 and 75% indicated moderate heterogeneity, and a value >75% indicated high heterogeneity. Forest plots were used to graphically display the effect size of each study and the pooled estimates. A *P* < 0.05 was considered statistically significant.

## Results

### Literature Search and Characteristics of the Included Studies

**Figure 1:**
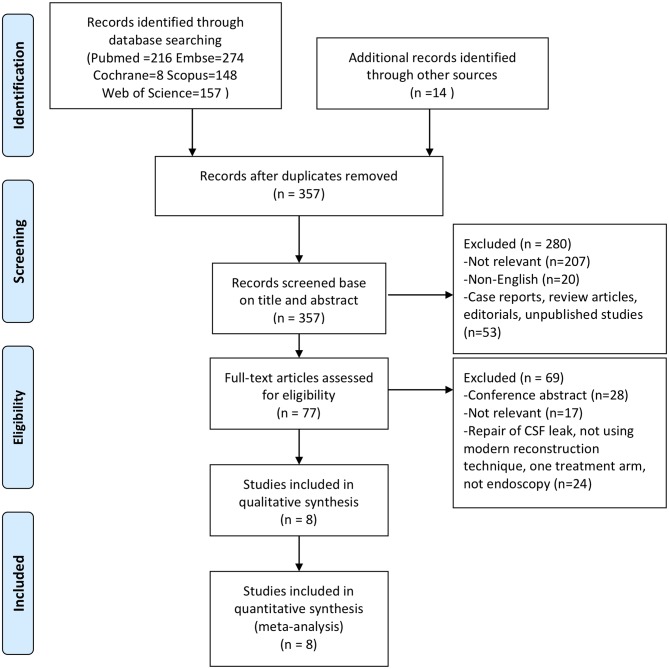
Preferred Reporting Items for Systematic Reviews and Meta-Analyses (PRISMA) flowchart summarizing the screening and selection process.

The search strategy identified a total of 357 studies after removing duplicates. Inclusion and exclusion criteria were applied to titles and abstracts of 357 articles. This yielded 77 studies that underwent full-text evaluation. Eight studies fulfilled the selection criteria and were included for quantitative analysis, as presented in the flow diagram (Figure 1). Demographic characteristics of these eight studies are summarized in Table 1. Tumor features and treatment strategies of included studies are summarized in Table 2. A total of 1,766 patients were considered suitable for this meta-analysis in these eight studies.

**Table 1:** Demographic characteristics of included studies.

**First author, year**	**Study design**	**Country**	**Sample size**	**Average age**	**Male (%)**	**BMI (kg/m^**2**^)**	**Risk of bias**
Patel et al. (6)	Retrospective cohort study	United States	146	NR	NR	NR	High quality^*^
Garcia-Navarro et al. (17)	Prospective cohort study	United States	46	53.3	NR	NR	High quality^*^
Ivan et al. (18)	Retrospective cohort study	United States	98	52	43.9%	BMI > 25, 75.5% BMI > 30, 41.8%	High quality^*^
Pereira et al. (19)	Prospective cohort study	United Kingdom	251	52	54.0%	NR	High quality^*^
Caggiano et al. (20)	Retrospective cohort study	United States	809	47.2	42.0%	BMI > 30, 32.7%	High quality^*^
Jonathan et al. (21)	Randomized control trials	India	60	39.2	51.7%	27.9 ± 5.9	High-risk of bias^†^
Zwagerman et al. (13)	Randomized control trials	United States	170	51.6	38.0%	28.1	Low-risk of bias^†^
Albarbi et al. (22)	Retrospective cohort study	Saudi Arabia	186	50.3	46.8%	NR	High quality^*^

* risk of bias was evaluated using The Newcastle–Ottawa Scale (NOS).

† risk of bias was evaluated using Cochrane risk of bias tool for RCTs.

*BMI, body mass index; NR, not reported*.

**Table 2:** Tumor features and treatment strategies of included studies.

**First author, year**	**Pituitary adenoma ratio (%)**	**Tumor location**			**Reconstruction strategy**	**LD placement criteria**	**LD protocol**
		**Anterior fossa**	**Sellar/** **suprasellar**	**Posterior fossa**			
Patel et al. (6)	NR	26	114	10	Multilayer reconstruction (NSF)	High-flow leakage	10 ml/h for 3 days
Garcia-Navarro et al. (17)	17.4%	NR	NR	NR	Multilayer reconstruction (NSF, gasket, fat, DuraSeal)	NR	5 ml/h for 1–2 days
Ivan et al. (18)	25.5%	36	24	26	Multilayer reconstruction (NSF, DuraGen, fat, DuraSeal)	NR	10–20 ml/h for 3–5 days
Pereira et al. (19)	75.3%	–	250	–	Multilayer reconstruction (NSF, DuraSeal)	Giant tumor, large suprasellar extension	NR
Caggiano et al. (20)	67.7%	NR	NR	NR	Multilayer reconstruction (NSF, fat graft, fascia lata)	Extended approach	NR
Jonathan et al. (21)	100.0%	–	60	–	Multilayer reconstruction	Randomized	Drain for 5 days
Zwagerman et.al. (13)	11.8%	35	84	50	Multilayer reconstruction (NSF, fascia lata, fat graft)	Randomized	10 ml/h for 3 days
Albarbi et al. (22)	100.0%	–	186	–	Multilayer reconstruction (NSF)	High-flow leakage, intracranial hypertension, poor reconstruction	Drain for 2 days

*LD, lumbar drainage; NR, not report; NSF, nasoseptal flaps*.

One RCT included in this meta-analysis was judged as low risk of bias, and the other one was judged as high risk of bias according to the Cochrane risk of bias tool. Based on the quality assessment by NOS, all included observational studies were judged as high quality with a score of 7/9 or 6/9 (Supplementary Table 1).

### Meta-Analysis of Efficacy and Safety

**Figure 2:**
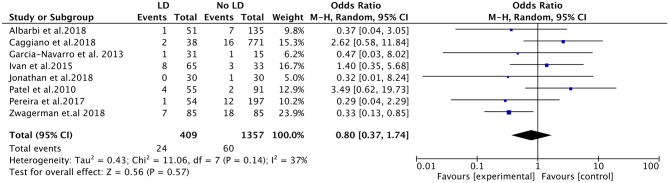
Forest plots showing odds ratio of post-operative cerebrospinal fluid (CSF) leakage in patients after endoscopic endonasal surgeries. CI, confidence interval; LD, lumbar drainage.

All of the eight studies evaluated the efficacy of LD placement in reducing risk of CSF leak by clinical evidence of CSF rhinorrhea. The overall post-operative CSF leak rate was 4.73% (84 cases). The post-operative leak rate was 5.87% when intraoperative LD was used, and the rate was 4.42% without LD placement. Among the eight studies, no significant difference was found in the post-operative leak rate between the LD group and the non-LD group (OR, 0.80; 95%CI, 0.37–1.74; *I*^2^ = 37%; *P* = 0.57) (Figure 2). There were three included studies that reported the AEs of LD (166 patients). The placement of LD was associated with increased risk of headache compared with the non-LD group (OR, 7.22; 95%CI, 1.23–42.29; *P* = 0.03; *I*^2^ = 0%). There was no statistically significant difference in the occurrence of deep vein thromboses and pulmonary emboli (OR, 1.44; 95%CI, 0.53–3.90; *P* = 0.48; *I*^2^ = 3%). In the total of 166 patients, one patient had a retained catheter that was observed without consequence.

### Subgroup Analysis

**Figure 3:**
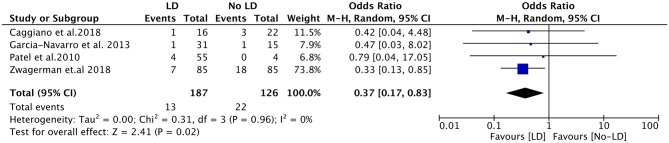
Forest plots showing odds ratio of post-operative cerebrospinal fluid (CSF) leakage in the setting of high-flow leaks. CI, confidence interval; LD, lumbar drainage.

Subgroup analyses were subsequently performed according to the flowrate of intraoperative leak, study design, and pathological type (Table 3). Intraoperative LD placement was associated with reduced likelihood of post-operative CSF leak in the setting of high-flow leaks (OR, 0.37; 95%CI, 0.17–0.83; *P* = 0.02; *I*^2^ = 0%; data available from 4 studies, 313 subjects) (Figure 3). Regarding the study design, the pooled OR for prospective studies showed a significant association between LD placement and decreased risk of CSF leak (OR, 0.34; 95%CI, 0.15–0.74; *P* = 0.007; *I*^2^ = 0%), whereas no significant difference was found in the retrospective studies (OR, 1.68; 95%CI, 0.73–3.90; *P* = 0.22; *I*^2^ = 5%). According to the ratio of pituitary adenomas, there was no significant difference between the four studies with a ratio of pituitary adenomas >60% (OR, 0.57; 95%CI, 0.21–1.52; *P* = 0.26; *I*^2^ = 29%) and the remaining three studies with a ratio of pituitary adenomas ≤ 60% (OR, 0.70; 95%CI, 0.19–2.53; *P* = 0.59; *I*^2^ = 33%).

**Table 3:** Subgroup analyses: intraoperative lumbar drainage in endoscopic endonasal skull base surgeries.

**Subgroup characteristics**	**Number of studies**	**Pooled OR (95% CI)**	***P***	**Heterogeneity**		
				***P***	***I*^**2**^**	**Chi^**2**^**
**Intraoperative CSF leaks**						
• High-flow leaks	4	0.37 (0.17, 0.83)	0.02	0.96	0%	0.31
Prospective vs. retrospective studies						
• Prospective studies	4	0.34 (0.15, 0.74)	0.007	1.00	0%	0.07
• Retrospective studies	4	1.68 (0.73, 3.90)	0.22	0.37	6%	3.18
Tumor type						
• Mixed	3	0.57 (0.21, 1.52)	0.26	0.25	29%	2.81
• Pituitary adenoma	4	0.70 (0.19, 2.54)	0.59	0.21	33%	4.50

*OR, odds ratio; CI, confident interval; RCTs, randomized controlled trials*.

### Sensitivity Analysis and Publication Bias

Sensitivity analyses were conducted by excluding one study at a time for each outcome. When we removed the study conducted by Zwagerman et al. (13), the heterogeneity decreased dramatically to 12%.

**Figure 4:**
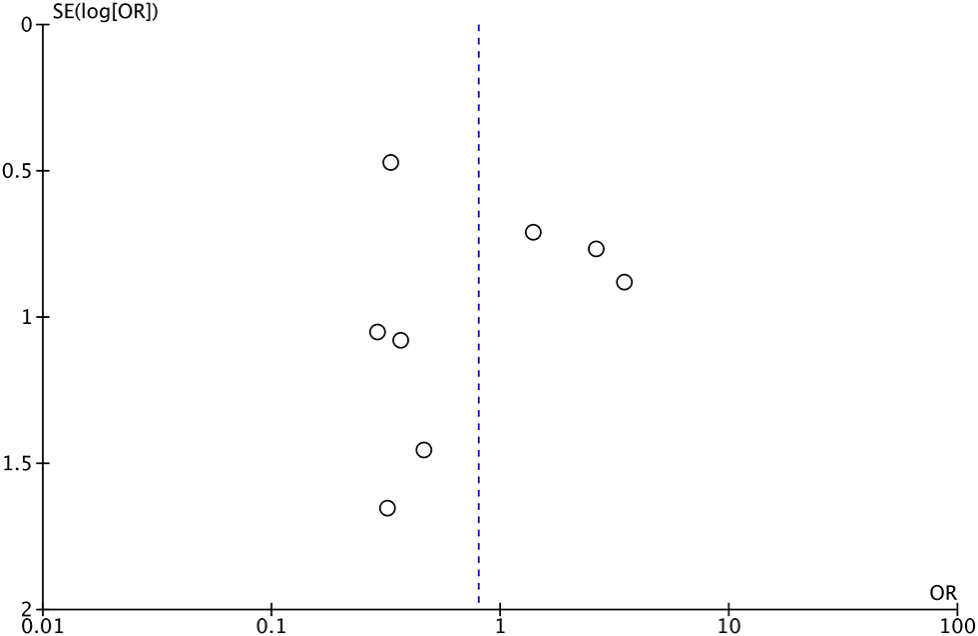
Funnel plots for publication bias. OR, odds ratio; SE, standard error.

Publication bias was tested using the data of LD placement and rate of CSF leak (*n* = 8). The shape of funnel plots showed no obvious asymmetry, which indicated the absence of significant heterogeneity between these selected studies (Figure 4).

## Discussion

This meta-analysis demonstrated that, in patients undergoing endoscopic skull base tumor resection, intraoperative LD placement was not significantly associated with a decreased risk of post-operative CSF leak. As for AEs, the LD placement was related to increased risk of headache, while no significant difference was observed in the occurrence of deep vein thromboses and pulmonary emboli.

These findings are in line with the previous meta-analysis that was based on only three studies (10). To the best of our knowledge, there were some limitations of that meta-analysis. First, the results relied on only three observational studies. Second, the included studies were of relatively poor quality, which may cause bias and confounding. Third, AEs were not assessed. Our present meta-analysis included recently published studies and examined the efficacy and safety of LD in patients undergoing endoscopic skull base surgery. Subgroup analyses were further performed according to the flowrate of intraoperative leak, study design, and pathological type.

Placement of intraoperative LD is often used for the purpose of providing a controlled, low-resistance egress of CSF during initial healing. To date, numerous studies have described various techniques to reduce the rate of post-operative CSF leak, including the use of multilayer closures with synthetic and autologous materials, the NSFs, the gasket seal, and Foley balloon (17, 23–25). Some studies reported that LD may not be needed in the endoscopic skull base tumor resection (3, 9). Tien et al. (26) published a systematic review on the management of post-operative CSF leaks in which they concluded that LD did not significantly contribute to successful repair in most low- or high-flow leaks. However, by analyzing the results, it is unusual that the CSF leak rate was higher in patients with LD placement than those without. This might represent a patient selection that LD was more likely to be used in higher-risk cases. Some studies also suggested that LD was not necessary in all high-flow CSF leaks (26–28). They reported 90–100% success rate from endoscopic repair without post-operative CSF diversion (24, 27).

In the recent RCT conducted by Zwagerman et al. (13) high-flow patients were recruited and randomized to either LD or no drainage. They found that LD placement was associated with decreased risk of post-operative CSF leak. The CSF leak rate was, respectively, 8.2% in the LD group and 21.2% in the non-LD group. Eloy et al. (27) reported a higher success rate from endoscopic repair without LD placement, possibly because they defined high-flow leak as a “leak brisk enough to visualize egress of CSF without Valsalva”. However, generally, most clinicians agreed with the definition, “entering into an arachnoid cistern or ventricle” (6, 13, 17, 29). To investigate the relationship between intraoperative high-flow leaks and LD use, we extracted data from four studies that specified the flowrate of intraoperative leak and performed the subgroup analysis. The result indicated that there was a statistically significant difference between LD group (7.0%) and non-LD group (17.5%). Furthermore, another RCT conducted by Lavigne et al. (30) enrolled patients with high-flow leaks of the anterior or post-erior cranial fossa. Their conclusion further supported our findings. However, this study has been only published as a meeting abstract without detailed data.

A discrepancy was identified in some studies that included pituitary lesions in the same category as large skull base lesions, such as meningiomas and craniopharyngiomas. Most pituitary tumors are located in the sellar region without an arachnoid extension and should be analyzed as a separate category, despite some pituitary adenomas are large enough and their removal can result in high-flow CSF leaks. The subgroup analysis based on the ratio of pituitary adenoma was performed, indicating that CSF leaks were not associated with the pathological type of pituitary adenoma.

Reported complications of the LD include headache, nerve root irritation, meningitis, tension pneumocephalus, acute or delayed intracranial hypotension, and subdural hemorrhage (3, 11). In our analysis, although serious complications were not observed in the total 166 patients, the risk of post-operative headache increased when the LD was placed (Table 4). In addition, there was one patient who suffered from a retained catheter without consequence, and it indicated that LD placement was associated with potential risk of reoperation. Some studies reported that LD placement was associated with an additional 2.0–3.2 days in the hospital (20, 22). Indeed, LD was left in place only for 1–3 days in most of our included studies (Table 2). Another aspect was that LD placement was associated with aforementioned patient selection (giant tumor, large suprasellar extension, and poor reconstruction). This may be a potential confounding factor affecting the length of stay. As for meningitis, several studies indicated no significant association between LD placement and post-operative infection or meningitis (5, 13, 21). In conclusion, the risks of LD placement should not be dismissed, and for those carefully selected, high-flow leak patients, the benefits of LD outweigh the risks.

**Table 4:** Pooled ORs of adverse events.

**Adverse events**	**Including study number**	**Pooled OR (95% CI)**	***P***	***I*^**2**^**
Headache	3	7.22 (1.23, 42.29)	0.03	0%
DVT and PE	2	1.44 (0.53, 3.90)	0.48	3%
Retained catheter	1	3.04 (0.12, 75.57)	0.50	NA

*OR, odds ratio; DVT, deep vein thromboses; PE, pulmonary emboli*.

Several limitations in this meta-analysis should be addressed. First, despite of rigorous eligibility criteria and a comprehensive search, the majority of included studies in this meta-analysis are observational studies that have inherent selection bias and confounders. In terms of generalizability due to larger and wider-spread samples, observational studies might be of value to explain the relationship between the LD placement and CSF leakage. Second, the RCT conducted by Jonathan et al. was judged as a high bias risk due to lack of blinding of the surgeons (21). High risk of bias may weaken confidence in the results. However, it was well-designed and met our inclusion criteria. Besides, only one in six domains met the criteria of high risk of bias. To make the result more convincing, we should include more studies with low risk of bias in the future. Third, a moderate degree of heterogeneity may limit our findings. On this point, we conducted the sensitivity analysis using the leave-one-out method. Heterogeneity decreased significantly from 37 to 12% after omitting studies conducted by Zwagerman et al. (13). Most included studies were observational studies, and only two of them were RCTs in our meta-analysis. As we know, RCTs have more strict study design and inclusion criteria than observational studies. The RCT conducted by Zwagerman et al. (13) only recruited patients with high-flow leaks; maybe this was the source of heterogeneity. Although the heterogeneity decreased the RCT conducted by Zwagerman et al. (13) was when removed, the heterogeneity (37%) of including all studies was also acceptable. Hence, this study should be included. Finally, subgroup analyses of the materials used in repair was not conducted due to limited data. This may be another confounder for this analysis. More studies with detailed evidence are needed to confirm the relationship.

This meta-analysis provides the up-to-date evidence, which has implications for clinical decision-making. This finding supports the importance of LD placement in the setting of high-flow leaks for the prevention of post-operative CSF leaks after endoscopic skull base tumor resection. Neurosurgeons should assess the benefits of LD placement and set these against the risks.

## Conclusion

Our meta-analysis provides evidence for efficacy and safety of intraoperative LD placement in preventing CSF leaks after endonasal skull base tumor resection and reconstruction. In the setting of intraoperative high-flow leaks, LD decreases the incident rate of CSF leak. Based on current evidence, LD is not recommended in all patients undergoing endoscopic skull base tumor resection.

## Data Availability Statement

All datasets generated for this study are included in the article/Supplementary Material.

## Author Contributions

XG and YH: development of methodology. XG and YZ: acquisition of data, analysis, and interpretation of data. XG: writing of the original manuscript. YH: revision of the manuscript.

## Conflict of Interest

The authors declare that the research was conducted in the absence of any commercial or financial relationships that could be construed as a potential conflict of interest.
