# Supplementary material for: Efficacy and Safety of Intraoperative Lumbar Drain in Endoscopic Skull Base Tumor Resection: A Meta-Analysis
Source: Front Oncol. 2020 May 7;10:606. doi: 10.3389/fonc.2020.00606 (PMC7221155; doi:10.3389/fonc.2020.00606)
Supplement: Supplementary file 1 [file Table_1.DOCX]

Pubmed

((lumbar AND drain) OR (CSF AND diversion)) AND (endonasal OR (skull AND base))

Cochrane

((lumbar AND drain) OR (CSF AND diversion)) AND (endonasal OR (skull AND base))

Embase

(lumbar AND ('drain'/exp OR drain) OR (('CSF'/exp OR CSF) AND ('diversion'/exp OR diversion))) AND (endonasal OR (('skull'/exp OR skull) AND ('base'/exp

OR base)))

Scopus

( ( ( lumbar AND drain ) OR (CSF AND diversion ) ) AND ( endonasal OR ( skull AND base ) ) )

Web of science

(((lumbar AND drain) OR (CSF AND diversion)) AND (endonasal OR (skull AND base)))
