# Supplementary material for: Efficacy and Safety of Intraoperative Lumbar Drain in Endoscopic Skull Base Tumor Resection: A Meta-Analysis
Source: Front Oncol. 2020 May 7;10:606. doi: 10.3389/fonc.2020.00606 (PMC7221155; doi:10.3389/fonc.2020.00606)
Supplement: Supplementary file 2 [file Table_2.DOCX]

**Risk of bias assessment of RCT in the meta-analysis by the Cochrane risk of bias tool for RCTs**

| Author | Year | Study design | Selection bias | | Performance bias | Detection bias | Attrition bias | Reporting bias | Overall risk of bias |
| --- | --- | --- | --- | --- | --- | --- | --- | --- | --- |
|  |  |  | Random sequence generation | Allocation concealment | Blinding of participants and personnel* | Blinding of outcome assessment* | Incomplete outcome data* | Selective reporting |  |
| Zwagerman et.al | 2018 | Prospective randomized trial | L | L | L | L | L | L | Low |
| Jonathan  et al | 2018 | Prospective randomized trial | L | L | H | L | L | L | High |

**Risk of bias assessment of studies included in the meta-analysis by the Newcastle-Ottawa Scale**

| Author (Year) | Adequacy selection of cohort | | | | Comparability of studies | | Outcome assessment | | | Total NOS score |
| --- | --- | --- | --- | --- | --- | --- | --- | --- | --- | --- |
|  | Representati-veness of the exposed cohort | Selection of the non-exposed cohort | Ascertain-ment of exposure | Demonstration that outcome of interest was not present at start of study | Study control for high-flow leaks and tumor type | Additional factors; controlled for≥2 variables including BMI and tumor size | Assessment of outcome | follow-up long enough for outcomes to occur | Adequacy of follow up of cohorts |  |
| Patel et al.2010 | * | - | * | * | - | - | * | * | * | 6/9 |
| Garcia-Navarro et al. 2013 | * | * | * | * | - | - | * | * | * | 7/9 |
| Ivan et al.2015 | * | - | * | * | - | - | * | * | * | 6/9 |
| Pereira et al.2017 | * | - | * | * | - | - | * | * | * | 6/9 |
| Caggiano et al.2018 | * | - | * | * | - | - | * | * | * | 6/9 |
| Albarbi et al.2018 | * | - | * | * | - | - | * | * | * | 6/9 |

Abbreviations: NOS, the Newcastle-Ottawa Scale;
